# Novel Human Anti-PD-L1 mAbs Inhibit Immune-Independent Tumor Cell Growth and PD-L1 Associated Intracellular Signalling

**DOI:** 10.1038/s41598-019-49485-3

**Published:** 2019-09-11

**Authors:** Margherita Passariello, Anna Morena D’Alise, Annachiara Esposito, Cinzia Vetrei, Guendalina Froechlich, Elisa Scarselli, Alfredo Nicosia, Claudia De Lorenzo

**Affiliations:** 1 Department of Molecular Medicine and Medical Biotechnology, University of Naples “Federico II”, Via Pansini 5, 80131 Napoli, Italy; 2 Ceinge – Biotecnologie Avanzate s.c. a.r.l., via Gaetano Salvatore 486, 80145 Naples, Italy; 3 Nouscom srl, Via di Castel Romano 100, 00128 Rome, Italy; 4 European School of Molecular Medicine, University of Milan, Via Festa del Perdono 7, 20122 Milan, Italy; 5 Keires AG Bäumleingasse 18, CH-4051 Basel, Switzerland

**Keywords:** Target identification, Cancer, Molecular medicine

## Abstract

The novel antibody-based immunotherapy in oncology exploits the activation of immune system mediated by immunomodulatory antibodies specific for immune checkpoints. Among them, the programmed death ligand-1 (PD-L1) is of particular interest as it is expressed not only on T-cells, but also on other immune cells and on a large variety of cancer cells, such as breast cancer cells, considering its high expression in both ErbB2-positive and Triple Negative Breast Cancers. We demonstrate here that PD-L1_1, a novel anti-PD-L1 T -cell stimulating antibody, inhibits PD-L1-tumor cell growth also by affecting the intracellular MAPK pathway and by activating caspase 3. Similar *in vitro* results were obtained for the first time here also with the clinically validated anti-PD-L1 mAb Atezolizumab and *in vivo* with another validated anti-mouse anti-PD-L1 mAb. Moreover, we found that two high affinity variants of PD-L1_1 inhibited tumor cell viability more efficiently than the parental PD-L1_1 by affecting the same MAPK pathways with a more potent effect. Altogether, these results shed light on the role of PD-L1 in cancer cells and suggest that PD-L1_1 and its high affinity variants could become powerful antitumor weapons to be used alone or in combination with other drugs such as the anti-ErbB2 cAb already successfully tested in *in vitro* combinatorial treatments.

## Introduction

The novel antibody-based immunotherapy in oncology exploits the activation of immune system, such as the stimulation of T-cells against cancer, mediated by immunomodulatory antibodies^1^ specific for different Immune Checkpoints (IC).

Among them, the programmed death ligand-1 (PD-L1), also called B7 homolog 1 (B7-H1), is of particular interest as it is expressed not only on T-cells, but also on other immune cells and a large variety of cancer cells, thus playing multiple roles. Indeed, PD-L1 is a cell surface protein expressed on activated APC, T and B lymphocytes and other cells, and, together with PD-L2, is the natural ligand of programmed cell death receptor-1 (PD-1). The interaction of PD-L1 with PD-1 on activated T-cells results in immunosuppression and tumor immune escape^2^. Moreover, PD-L1 stimulates the proliferation of T_Reg_ cells, which also express high levels of PD-1^3^. This feature is exploited by the tumors as another mechanism to escape from immune surveillance, considering that many tumors are highly infiltrated with T_Reg_ cells suppressing effector immune responses^4^.

Since the interaction of PD-1, expressed on the surface of activated T-cells, and PD-L1, displayed on the surface of tumor cells, results in immunosuppression, the interference mediated by an antibody specific for PD-L1 is a suitable strategy to reduce the suppression of effector T-cells.

To date, a number of antibodies targeting PD-L1 are in clinical use or development for the therapy of cancer: human or humanized mAbs targeting the immunosuppressive receptor PD-L1, such as Atezolizumab, Durvalumab and Avelumab, have been approved for the treatment of several tumors, including melanoma, non-small cell lung cancer, renal cell carcinoma, urothelial carcinoma, liver carcinoma, microsatellite instable (MI) colorectal cancer and Merkel-cell carcinoma^5–7^.

Interestingly, high levels of PD-L1 have been recently reported also on breast cancer cells and a high proportion of PD-L1-positive tumors has been found co-infiltrated with PD-1^+^-lymphocytes^8^. Notably, Triple-negative breast cancer (TNBC) specimens showed the highest level of PD-L1 expression, followed by HER2 overexpressing subtypes.

We recently generated a novel human anti-PD-L1 mAb, called PD-L1_1, which was found to have the capacity to interfere in the PD-L1/PD-1 interaction, to strongly activate T-cell proliferation and to induce cytokine secretion more efficiently than the clinically validated antibodies Nivolumab and Atezolizumab^9^. This antibody cross-reactive with murine PD-L1, was found to be effective also *in vivo* for its antitumor activity on mice bearing colon cancer but it was not tested yet for its efficacy on human mammary tumor cells.

Noteworthy, the immune system plays a crucial role in the outcomes of some BC subgroups of patients, especially more aggressive, proliferative ones such as triple-negative and HER2-positive BC [8]. Hence, PD-L1/PD-1-axis could be a useful therapy target for both tumor entities, in order to avoid the tumor escape from the immunological defence^10^.

Furthermore, PD-L1 seems to play not only a role in the interaction with PD-1 on lymphocytes, but also by itself on tumor cells by inducing cell proliferation, as it has been reported in literature that PD-L1 expression increases the levels of Ki-67 and other proteins involved in tumor cell proliferation, thus suggesting that it could become a marker of tumor aggressiveness^11^.

Moreover, Massi *et al*. studied the intracellular pathways in stable PD-L1^**+**^ and PD-L1^**−**^ subpopulations of a tumor cell line, and found that the PD-L1^+^ cells showed a constitutively higher degree of phosphorylation of ERK1/2, p38 and JNK, compared to that observed in PD-L1^**−**^ cells^12^.

These proteins are responsible for the transduction of extracellular signals into the cells. In particular, P38 and JNK are responsive to stress stimuli and regulate several important cellular functions including cell growth, differentiation, survival and apoptosis^13,14^, whereas Erk plays an important role in MAPK pathway and promotes cell growth and proliferation in many mammalian cell types^15^.

Thus, PD-L1 expression on tumor cells could be considered as a predictive marker of enhanced aggressiveness and invasiveness, however further studies are still needed to clarify the mechanism of action of PD-L1 and its associated pathways.

Since both ErbB2-positive tumors and TNBC express high levels of PD-L1^16,17^ and a high proportion of PD-L1 positive tumors are co-infiltrated with PD1^+^ infiltrating lymphocytes^18^, a new therapeutic approach could also include combinations of anti-ErbB2 or anti-EGFR drugs with the novel anti-PD-L1 mAbs. This approach is already under investigation for a combination of the anti-ErbB2 Trastuzumab and anti-PD1 Pembrolizumab^8^, and could be further adopted in the future considering that some immunomodulatory antibodies have already been used in clinical trials for the therapy of breast cancer showing safety and efficacy^8,16,17^. Indeed, Atezolizumab has been tested in monotherapy in the phase I clinical trial in a cohort of 116 metastatic heavily pretreated TNBC patients^19^, and found to be effective and well tolerated.

Furthermore, recently the combination of Atezolizumab and nab-paclitaxel versus placebo and nab-paclitaxel has been investigated in the IMpassion130 phase III trial involving patients with locally advanced or metastatic triple-negative breast cancer^20^, and it was reported to provide long-term clinical benefit in metastatic TNBC.

Moreover Atezolizumab is currently in Phase IIA clinical trial assessing the safety and efficacy in combination with paclitaxel, Trastuzumab, and Pertuzumab in 50 patients with locally advanced, unresectable, or metastatic HER2-overexpressing breast cancer (ClinicalTrials.gov Identifier:NCT03125928).

Thus, considering PD-L1 as a promising target for breast cancer, we investigated the ability of PD-L1_1, the novel human anti-PD-L1 antibody^9^ and its high affinity variants (Cembrola *et al*., Rapid affinity maturation of novel anti-PD-L1 antibodies by a fast drop of the antigen concentration and FACS selection of yeast libraries, submitted for publication, 2019), previously generated in our laboratory, to inhibit PD-L1-positive mammary tumor cell growth.

Furthermore, in order to clarify the role of PD-L1 in breast tumor cell growth and proliferation, we investigated the effects of Atezolizumab and the novel human anti-PD-L1 antibodies generated in our laboratory, on the intracellular pathways associated with PD-L1.

Finally, to verify whether combinatorial treatments of anti-ErbB2 and anti-PD-L1 antibodies can potentiate their antitumor effects, PD-L1_1 and an anti-ErbB2 compact antibody (Erb-hcAb) capable of inhibiting breast tumor growth, previously developed in our laboratory^21–24^, have been tested in combination to evaluate by cytotoxic assays their effects on tumor growth in comparison with the single treatments.

## Results

### Binding of PD-L1_1 mAb to breast cancer cells expressing PD-L1

Many ongoing clinical trials in breast cancer patients are evaluating immunotherapy based on immunomodulatory antibodies^8,25^.

Since PD-L1 plays a critical role in breast cancer, as it is expressed at high levels by both ErbB2-positive and TNBC, and a high proportion of PD-L1-positive tumors are infiltrated with PD-1-positive lymphocytes, it can be considered as a potential target for breast cancer treatments^26,27^.

**Figure 1:**
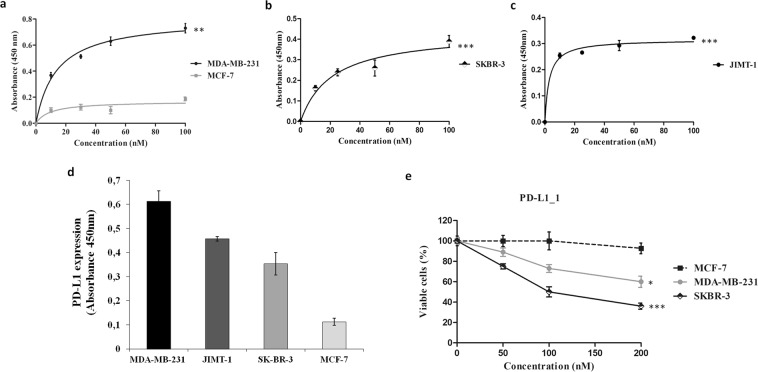
Binding curves and anti-tumor effects of PD-L1_1 mAb on breast cancer cells. The binding curves were obtained by ELISA assays of PD-L1_1 tested at increasing concentrations on MDA-MB-231 (**a**), SK-BR-3 (**b**) and JIMT-1 (**c**) cells. MCF-7 cells were used in parallel assays as a negative control (grey curve, panel a). The expression of PD-L1 on the indicated tumor cell lines was tested by cell ELISA with a commercial anti-PD-L1 antibody (**d**). Effects of PD-L1_1 on MDAMB231 (gray curve), SK-BR-3 (black curve) or MCF-7 cells(dashed line). Cells were treated for 72 h with PD-L1_1 mAb tested at increasing concentrations and cell survival was expressed as percentage of viable cells with respect to untreated cells (**e**). Error bars depicted means ± SD. P values for the indicated treatment relative to untreated cells, are: ***P ≤ 0.001; **P < 0.01; *P < 0.05.

Due to the ability of the novel isolated PD-L1_1 mAb to recognize with high affinity and specificity PD-L1, not only as a purified protein but also on human T-cells^9^, we tested its binding to PD-L1 also on breast tumor cells. To this aim, we used both TNBC MDA-MB-231 cells (Fig. 1a), and ErbB2-positive breast cancer SK-BR-3 and JIMT-1 cell lines (Fig. 1b,c) expressing satisfactory levels of PD-L1 (Fig. 1d) on the cell membrane^28,29^. The MCF-7 mammary cell line, expressing low levels of cell surface PD-L1 (see Fig. 1d), was used as a negative control (Fig. 1a). As shown, PD-L1_1 selectively binds to PD-L1-positive tumor cells with affinities comparable to those previously observed on lymphocytes^9^, whereas only a poor binding was observed on MCF-7 cells, thus confirming its binding specificity for the target cells with a positive correlation between the level of expression of PD-L1 on the cells and the extent of binding of PD-L1_1 to those cells.

### Biological activity of PD-L1_1 on breast cancer cells

Since PD-L1_1 is able to recognize with high affinity and specificity PD-L1 expressed not only on T-cells but also on breast cancer cells, we decided to investigate the *in vitro* effects of PD-L1_1 on breast tumor cells. To this aim, PD-L1_1 was tested at increasing concentrations (50–200 nM) on mammary SK-BR-3 and MDA-MB231 cells for 72 hours at 37 °C in the absence of lymphocytes. As a control, PD-L1_1 was also tested in parallel, in the same conditions, on PD-L1-negative MCF-7 breast cancer cells. As shown in Fig. 1e, PD-L1_1 significantly inhibited the growth of both the PD-L1-positive cell lines in a dose dependent-manner, whereas no effects were observed on the viability of MCF-7 cells, thus confirming the specificity of its biological effects.

**Figure 2:**
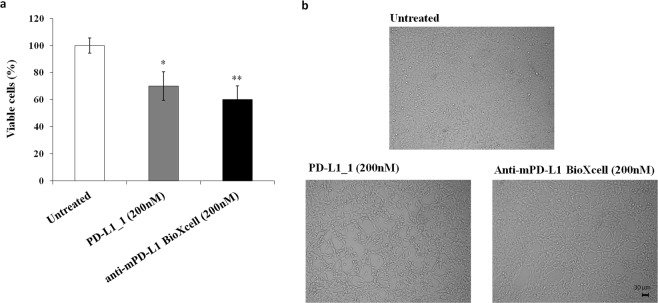
Effects of the anti-PD-L1 mAbs on the viability of CT26 colon cancer cells. Effects of PD-L1_1 (grey bar) or anti-mouse PD-L1 (black bar) BioXcell mAb on CT26 colon cancer cells. Cells were treated for 72 h with the anti-PD-L1 mAbs tested at the concentration of 200 nM and cell survival was expressed as percentage of viable cells with respect to untreated cells (**a**). Representative images of CT26 cells treated as indicated (**b**). The untreated cells were used as a negative control. Error bars depicted means ± SD. P values for the indicated mAbs relative to untreated cells, are: **P < 0.01, *P < 0.05. Scale bar = 30 μm.

Furthermore, the antitumor activity of PD-L1_1 was also tested in comparison with that of an anti-mouse PD-L1 (clone 10F.9G2, BioXcell) on mouse CT26 colon cancer cells. They were both found able to inhibit cell viability of about 30–40% at a concentration of 200 nM (see Fig. 2), thus indicating that the antitumor effect of PD-L1–1 was exerted not only on mammary cancer cells but also on different types of tumor cells.

**Figure 3:**
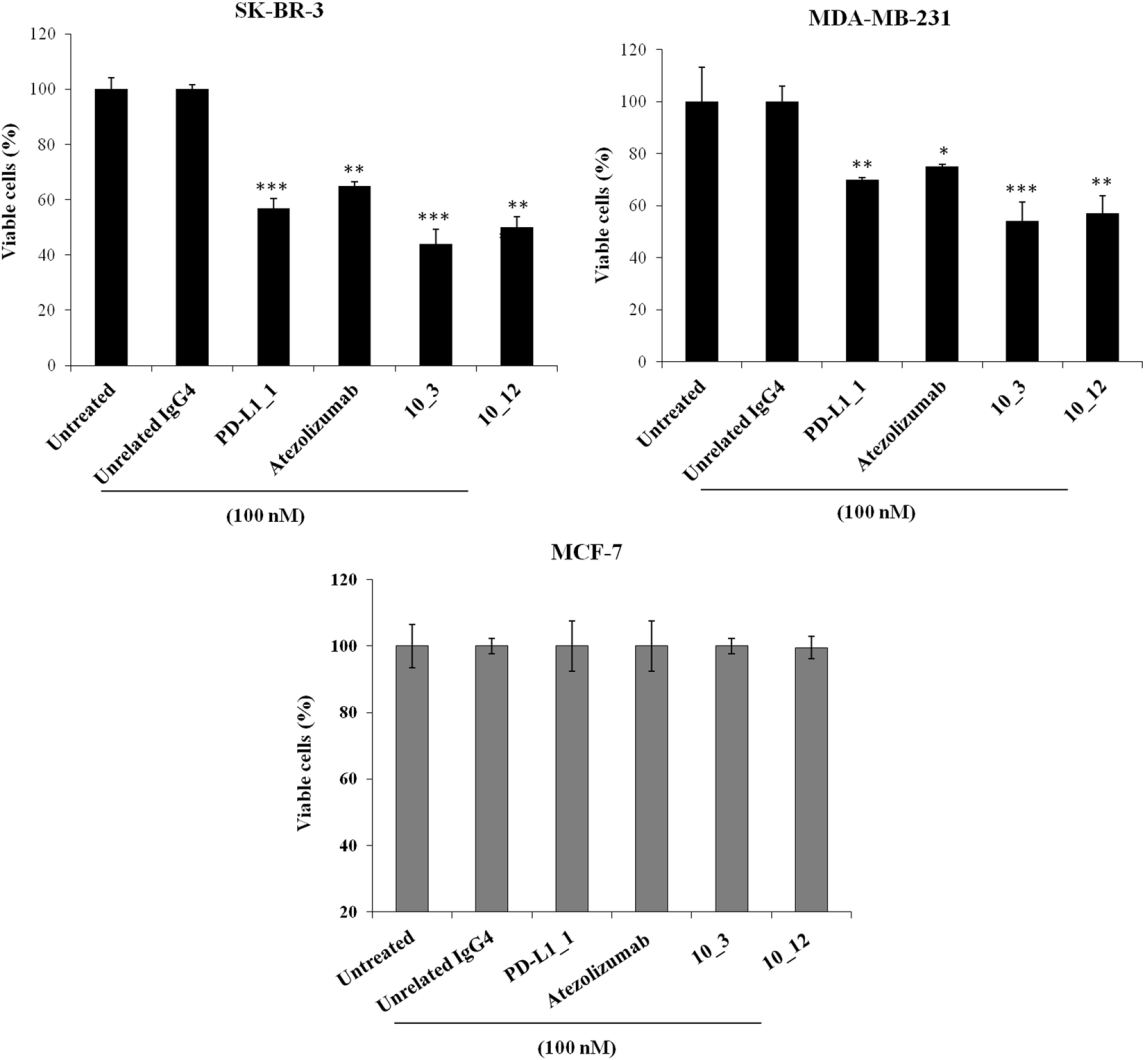
Anti-tumor effects of PD-L1_1 and its derived high affinity variants 10_3 and 10_12 on breast cancer cells. SK-BR-3, MDA-MB-231 and MCF-7 cells were incubated for 72 hours at 37 °C with PD-L1_1, 10_3 or 10_12 mAbs, at the indicated concentration. Cell survival is expressed as percent of viable treated cells with respect to untreated cells. Error bars depicted means ± SD. P values for the indicated mAbs relative to unrelated IgG4 mAb, are: ***P ≤ 0.001; **P < 0.01; *P < 0.05.

**Figure 4:**
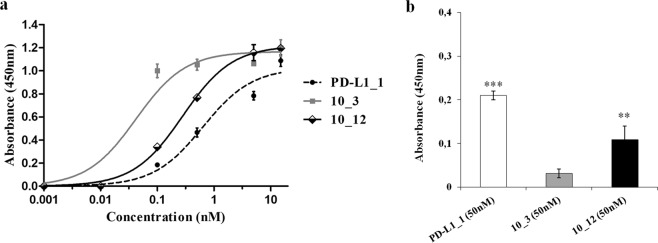
Binding affinity of PD-L1_1 and its variants to human PD-L1 protein and mouse CT26 cancer cells. (**a**) ELISA assay on human PD-L1/Fc protein carried out with 10_3 (grey curve), 10_12 (black curve) or PD-L1_1 (dashed curve). (**b**) Cell ELISA on CT26 colon cancer cells to test the crossreactivity of 10_3 (grey bar) and 10_12 (black bar) mAbs compared to that of the parental PD-L1_1 (white bar). Error bars depicted means ± SD. P values for the indicated mAbs relative to untreated control, are: ***P ≤ 0.001; **P < 0.01.

In order to compare the biological anti-tumor activity of PD-L1_1 with that of the clinically validated anti-PD-L1 mAb Atezolizumab, we tested them in parallel at the dose of 100 nM on the indicated breast cancer cells (Fig. 3 and Supplementary Fig. S1), by including an unrelated IgG4 isotype antibody as a negative control. As a further positive control, two variants of PD-L1_1 with higher affinity for PD-L1, called 10_3 and 10_12 (Cembrola *et al*., Rapid affinity maturation of novel anti-PD-L1 antibodies by a fast drop of the antigen concentration and FACS selection of yeast libraries, submitted for publication, 2019) were tested in parallel assays. These affinity-matured anti-PD-L1 antibodies were obtained by yeast surface display FACS-based methodology coupled with a CDR-targeted mutagenesis protocol applied to a single CDR in the heavy chain of PD-L1_1. The best variant 10_3 and 10_12 IgGs show lower equilibrium dissociation constants (about 10 fold lower K_D_) with PD-L1 than the wild type one (see Fig. 4). Accordingly, they were found able to strongly inhibit the growth of both the tumor cell lines, with effects even more potent than those of the parental PD-L1_1 and Atezolizumab. When tested on MCF-7 cells, used as a negative control, PD-L1_1 and its high affinity variants did not show significant effects (Fig. 3 and Supplementary Fig. S1), as expected.

Unfortunately, 10_3 and 10_12 did not show the anti-tumor effects of the parental PD-L1_1 when tested on CT26 cancer cells due to unexpected loss of their binding ability to these cells (see Fig. 4), likely caused by mutations occurred in the affinity maturation process (Cembrola *et al*., Rapid affinity maturation of novel anti-PD-L1 antibodies by a fast drop of the antigen concentration and FACS selection of yeast libraries, submitted for publication, 2019).

### Effects of anti-PD-L1 mAbs on intracellular pathways downstream PD-L1

Considering the *in vitro* anti-tumor effects of the novel isolated anti-PD-L1 mAb and its high affinity variants on breast cancer cells we made the hypothesis that PD-L1 may play by itself a role on tumor cells, by inducing cell proliferation, and anti-PD-L1 mAbs might inhibit its effects.

To test this hypothesis on the role of PD-L1, we firstly used PD-1/Fc fusion protein as an agonist, to activate PD-L1 and eventually tumor cell growth, and IFN-γ to inhibit cell growth and induce apoptosis^10,30^.

After the treatments of SK-BR-3 cells with PD-1/Fc (1 µg/ml) or IFN-γ (100 ng/ml), carried out for 72 hours at 37 °C, we analysed the effects on both tumor cell survival and on the pathways downstream PD-L1.

As shown in Supplementary Fig. S2b, we observed an increase of cell proliferation when the tumor cells were treated in the presence of PD-1/Fc and a significant reduction of the cell viability when they were treated with IFN-γ, accordingly with similar effects of IFN-γ on other tumor cell lines previously reported^28,30–35^. As a negative control, these experiments were repeated on PD-L1-negative MCF-7 cells and no significant effects were observed (Supplementary Fig. S2a).

In parallel, by western blotting analyses we evaluated the level of phosphorylated Erk, which was reported to play a role as a promoter of tumor cell proliferation, and Cleaved caspase-3, as a marker of apoptosis^14,15^. As shown in Supplementary Fig. S2c, the level of p-Erk increased in the presence of PD-1/Fc, whereas the level of Cleaved caspase-3 significantly decreased compared to untreated cells. On the contrary, when the cells were treated with IFN-γ, the level of p-Erk was reduced, whereas the level of Cleaved caspase-3 was higher compared to untreated cells.

These results suggest that PD-L1, upon binding to its receptor PD-1, induces the phosphorylation of Erk and inhibits tumor cell death, in line with similar results observed in a PD-L1+ cell line, and previously reported^11,12^.

Indeed, PD-L1 has been reported in literature to affect tumor cell proliferation by increasing the levels of p-Erk, p-JNK and p-P38 proteins, which are members of the MAPK family and play an important role in the transduction of extracellular signalling, thus regulating several cellular functions such as cell proliferation, survival and differentiation^13–15^.

**Figure 5:**
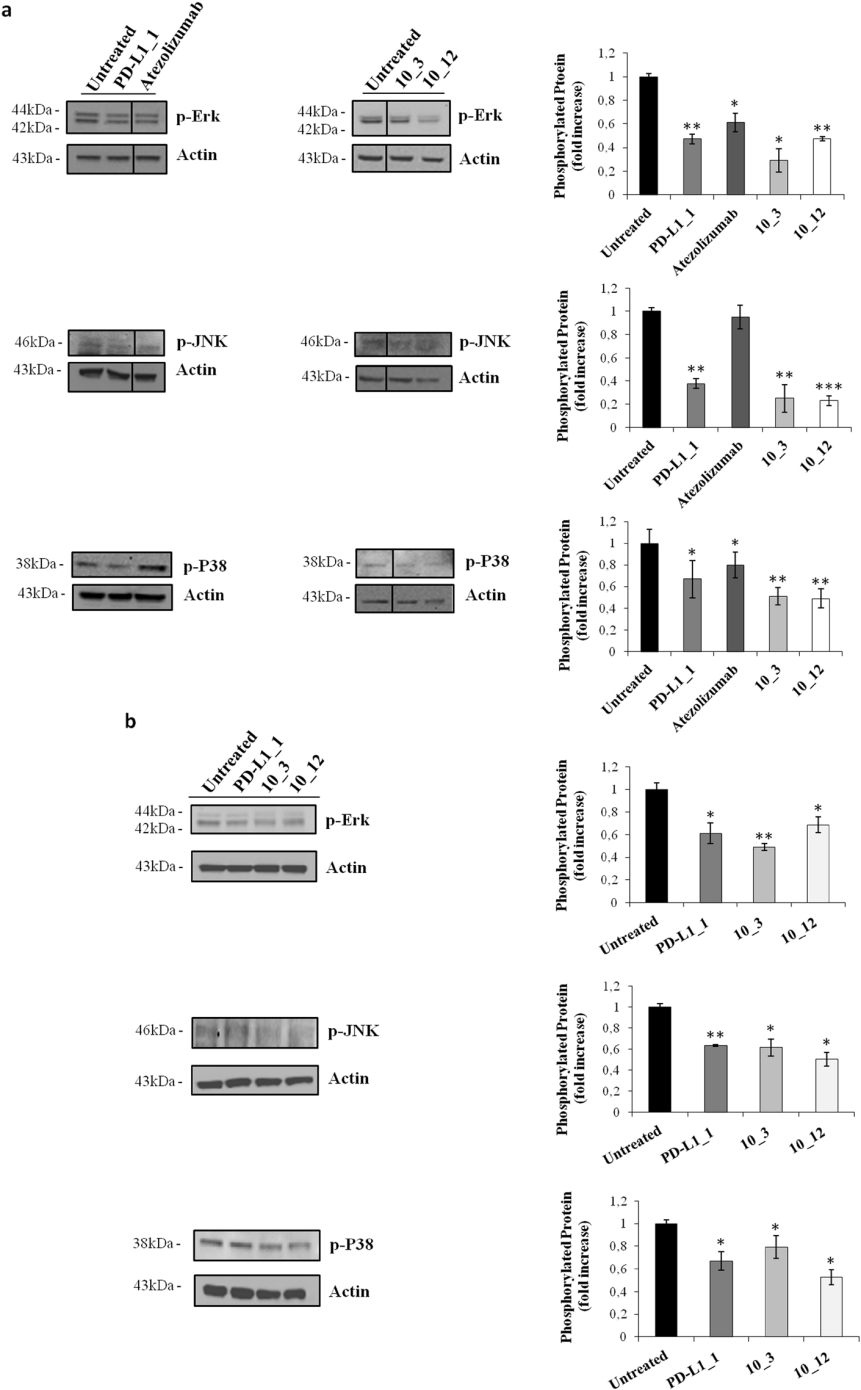
Effects of PD-L1_1 on the pathways associated with PD-L1 in tumor cells. Western blotting analyses with the specific antibodies for the indicated proteins of extracts from SK-BR-3 (**a**) and MDA-MB-231 (**b**) tumor cells treated in the absence or in the presence of PD-L1_1 or its high affinity variants. Atezolizumab was used as a positive control. A line has been inserted to indicate distant lanes of the same gel (see Supplementary dataset). Protein levels are also expressed as fold increase with respect to those observed in untreated cells and normalized to actin. Error bars depicted means ± SD. P values for the indicated mAbs relative to untreated cells, are: ***P ≤ 0.001; **P < 0.01; *P < 0.05.

In order to test whether the PD-L1_1 antibody inhibits tumor cell proliferation by similarly affecting these pathways, we performed western blotting analyses of extracts from breast SK-BR-3 and triple negative MDA-MB-231 tumor cells treated for 72 hours at 37 °C in the absence or in the presence of PD-L1_1, used at the concentration of 200 nM. As shown in Fig. 5a and in Fig. 5b, the level of phosphorylated Erk, P38 and JNK proteins significantly decreased when both SK-BR-3 and MDA-MB-231 cells were treated with PD-L1_1 compared to untreated cells. No significant effects were observed on the level of total Erk, P38 and JNK (data not shown), as well as no significant effects were observed on both the levels of p-Akt and total Akt (see Supplementary Fig. S3).

Atezolizumab, used as a positive control on SK-BR-3 tumor cells, showed similar effects on p-Erk, but, differently from PD-L1_1, did not affect the level of p-JNK, and showed only a slight effect on p-P38 (Fig. 5a). As additional controls, the two high affinity variants of PD-L1_1, 10_3 and 10_12, were tested in parallel assays on SK-BR-3 or MDA-MB-231 tumor cells. After treatments with 10_3 and 10_12, the phosphorylation levels of Erk, P38 and JNK were found to be strongly reduced by these variants (Fig. 5a,b), that showed a more potent effect compared to the parental PD-L1_1 mAb in both tumor cell lines. However, the effects obtained with the two variants of PD-L1_1 were more marked on SK-BR-3 cells compared to those observed on MDA-MB-231 cells, in line with the results described above on tumor cell viability, in which 10_3 and 10_12 inhibited tumor cell growth of SK-BR-3 tumor cells more efficiently compared to MDA-MB-231 tumor cells.

Thus, the anti-PD-L1 antibodies seem to stress tumor cells and inhibit their cell proliferation supporting the idea that PD-L1 could represent a marker for cancer, independent from the immune system. Furthermore we show here for the first time that the intracellular pathways downstream PD-L1 involving MAPKs, are inhibited by anti-PD-L1 mAbs, even though further studies are still needed to clarify their mechanisms of action.

**Figure 6:**
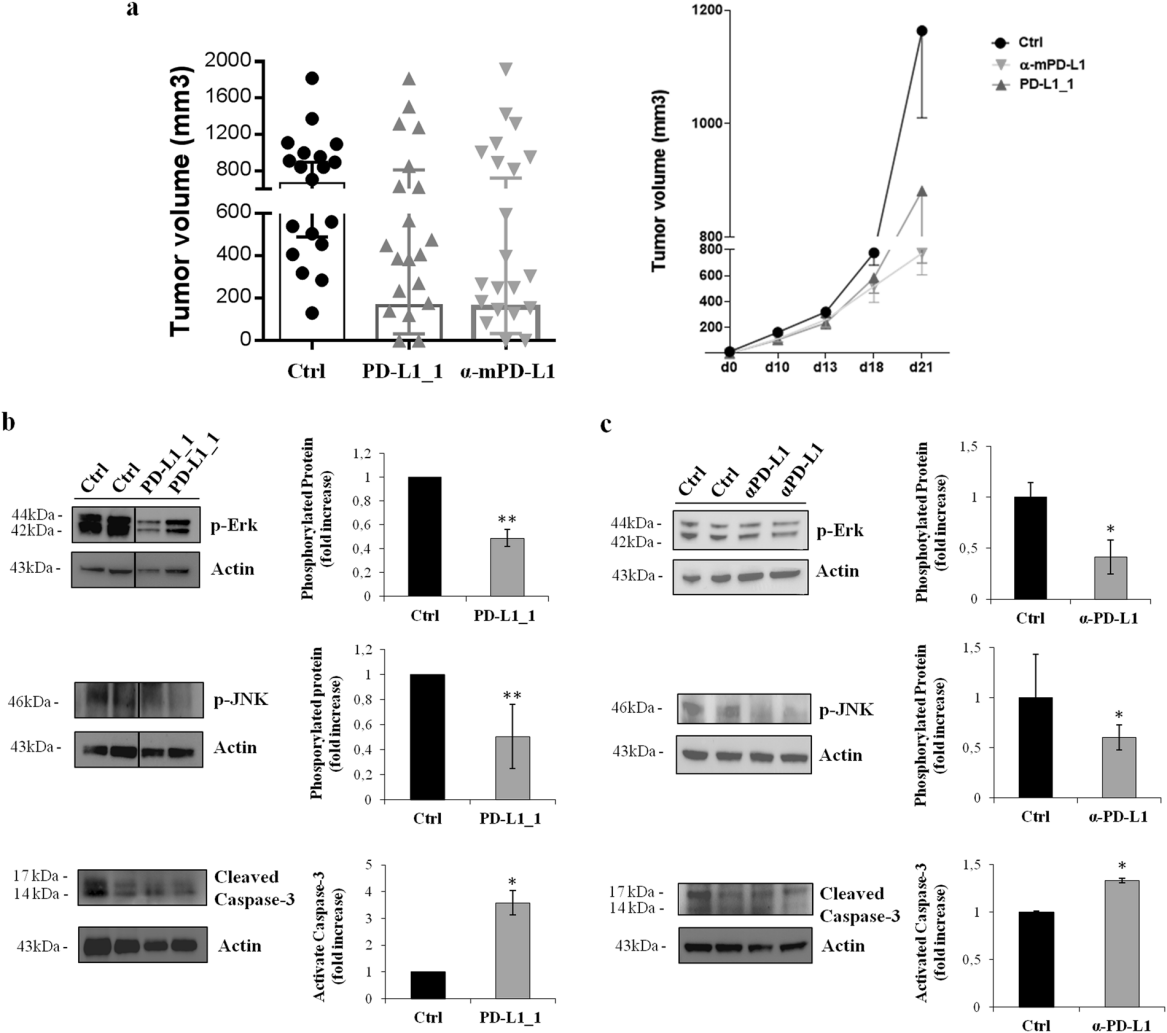
Effects of anti-PDL1 mAbs on tumor growth and signal transduction in CT26 tumors *in vivo*. (**a**) Tumor growth in mice inoculated sc with CT26 cells at day 0 and left untreated (black) or treated with PD-L1_1 (grey) or α-mPD-L1 (light grey). Shown is tumor volume for individual mice (n = 20) at day 21 (left panel) and mean of tumor volume for each group over time (right panel), *p = 0.02. Western blotting was performed on cell extracts from tumor specimens of two mice killed on day 21 from untreated groups or from responder groups treated with PD-L1_1 (**b**) or with α-mPD-L1 (**c**), as described in Methods. A line has been inserted to indicate distant lanes of the same gel (see Supplementary dataset). Protein levels are also expressed as fold increase with respect to those observed in untreated mice and normalized to actin. Error bars depicted means ± SD. P values for the indicated mAbs relative to cell extracts from untreated groups, are: ***P ≤ 0.001; **P < 0.01; *P < 0.05.

To confirm this hypothesis on the role of PD-L1 on tumor cells, we tested also the effects of PD-L1_1 and a commercially available anti-mouse PD-L1 mAb (clone 10F.9G2, BioXcell), previously validated *in vivo*^36^, on these pathways in PD-L1-positive colon CT26 tumors *in vivo*. To this aim, mice were implanted with CT26 cells (day 0) and then treated with PD-L1_1 or anti-mouse PD-L1 antibody (200 µg ip, clone 10F.9G2, BioXcell) reacting against murine PD-L1 (day 3, 6, 10). While the growth rate of tumors in untreated mice was very fast and uncontrolled, with the majority of tumors reaching sizes of >650 mm^3^ at day 21, a drastic reduction in tumor volume was found in mice treated with α-mPD-L1 (p = 0.02), and similar effects were observed in mice treated with PD-L1_1, as expected^9^ (Fig. 6a). During the period of treatment, the animals did not show significant changes of weight or other visible signs of toxicity.

We then analyzed the effects of PD-L1_1 and α-mPD-L1 treatments on the activation of MAPK proteins having a critical role in cancer cell proliferation and found to be involved in PD-L1 intracellular signaling. Western blotting analyses were performed on cell extracts from tumors removed at the end of the experiment on day 21, and processed as described in Methods. As shown in Fig. 6b and in Fig. 6c, the anti-PD-L1 antibodies inhibited the phosphorylation/activation of MAPK and JNK and induced the cleavage of caspase-3 more efficiently compared to untreated tumor cells, thus confirming the results relative to the association of PD-L1 to these pathways observed *in vitro*, and mentioned above.

### Antitumor effects of PD-L1_1 combined with anti-ErbB2 antibody

It has been reported that combinatorial treatments of anti-PD-L1 with anti-ErbB2 drugs can lead to more potent effects as compared to treatment with each single agent^8^ (ClinicalTrials.gov Identifier:NCT03125928).

Due to the *in vitro* anti-tumor efficacy of the novel isolated PD-L1_1 mAb and to its ability to recognize with high affinity and specificity PD-L1 also on breast cancer cells, inhibiting cell growth and affecting the downstream MAPK pathways, we investigated the possibility to use it in combination with the anti-ErbB2 compact antibody, Erb-hcAb, capable of inhibiting breast tumor cell growth *in vitro* and *in vivo*^21,24,37^.

**Figure 7:**
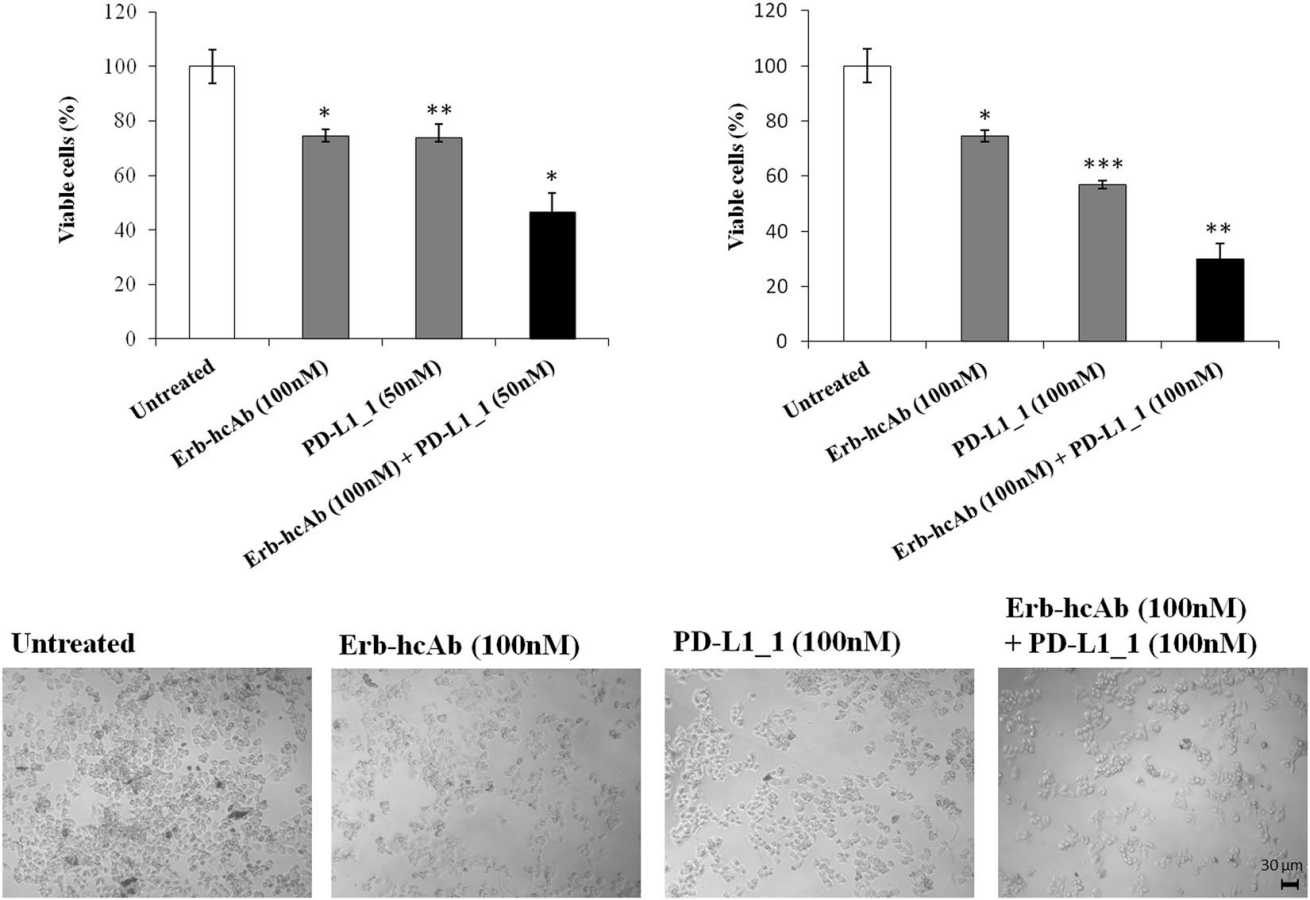
Combined treatments of Erb-hcAb and PD-L1_1 on SK-BR-3 tumor cells. SK-BR-3 cells were treated for 72 hours with Erb-hcAb or PD-L1_1 mAb, alone or in combination, at the indicated concentrations. SK-BR-3 cell survival is expressed as percent of viable treated cells with respect to untreated cells. Images of SK-BR-3 cells untreated or treated with each indicated compound or with the indicated combination. Error bars depicted means ± SD. P values for the indicated mAbs relative to untreated cells, are: ***P ≤ 0.001; **P < 0.01; *P < 0.05. Scale bar = 30 μm

With this aim SK-BR-3 cells were treated for 72 hours at 37 °C with increasing concentrations (50–100 nM) of Erb-hcAb and PD-L1_1 mAbs, used alone or in combination. We show here that the combinatorial treatment inhibits the growth of tumor cells more efficiently than when they are used as single agents (Fig. 7). An unrelated IgG4 isotype control antibody was used as a negative control (data not shown).

**Figure 8:**
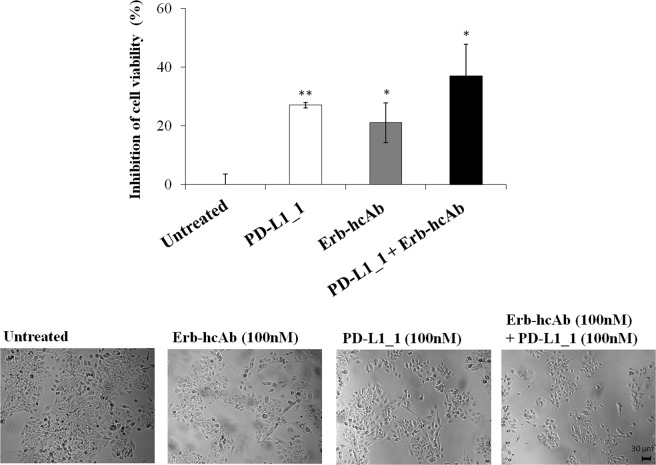
Combinatorial treatment of PD-L1_1 with Erb-hcAb on JIMT-1 tumor cells. Effects on tumor cell survival of Erb-hcAb and PD-L1_1, alone or in combination, at the indicated concentrations on JIMT-1 cell lines. Cell survival is expressed as percent of viable treated cells with respect to untreated cells. The images show some fields of cultures of cells untreated or treated with each indicated compound or with the indicated combination. Error bars depicted means ± SD. P values for the indicated mAbs relative to untreated cells, are: **P < 0.01; *P < 0.05. Scale bar = 30 μm.

To test whether the combinatorial treatment could be useful also for inhibiting the growth of more aggressive breast cancer cells resistant to Trastuzumab, we used JIMT-1 cells, derived from a metastasis of breast cancer patient, in a parallel assay. As shown in Fig. 8, the combinatorial treatment inhibited the growth of these tumor cells more efficiently than when they were used as single agents, proving to be beneficial also in Trastuzumab-resistant JIMT-1 cells.

**Figure 9:**
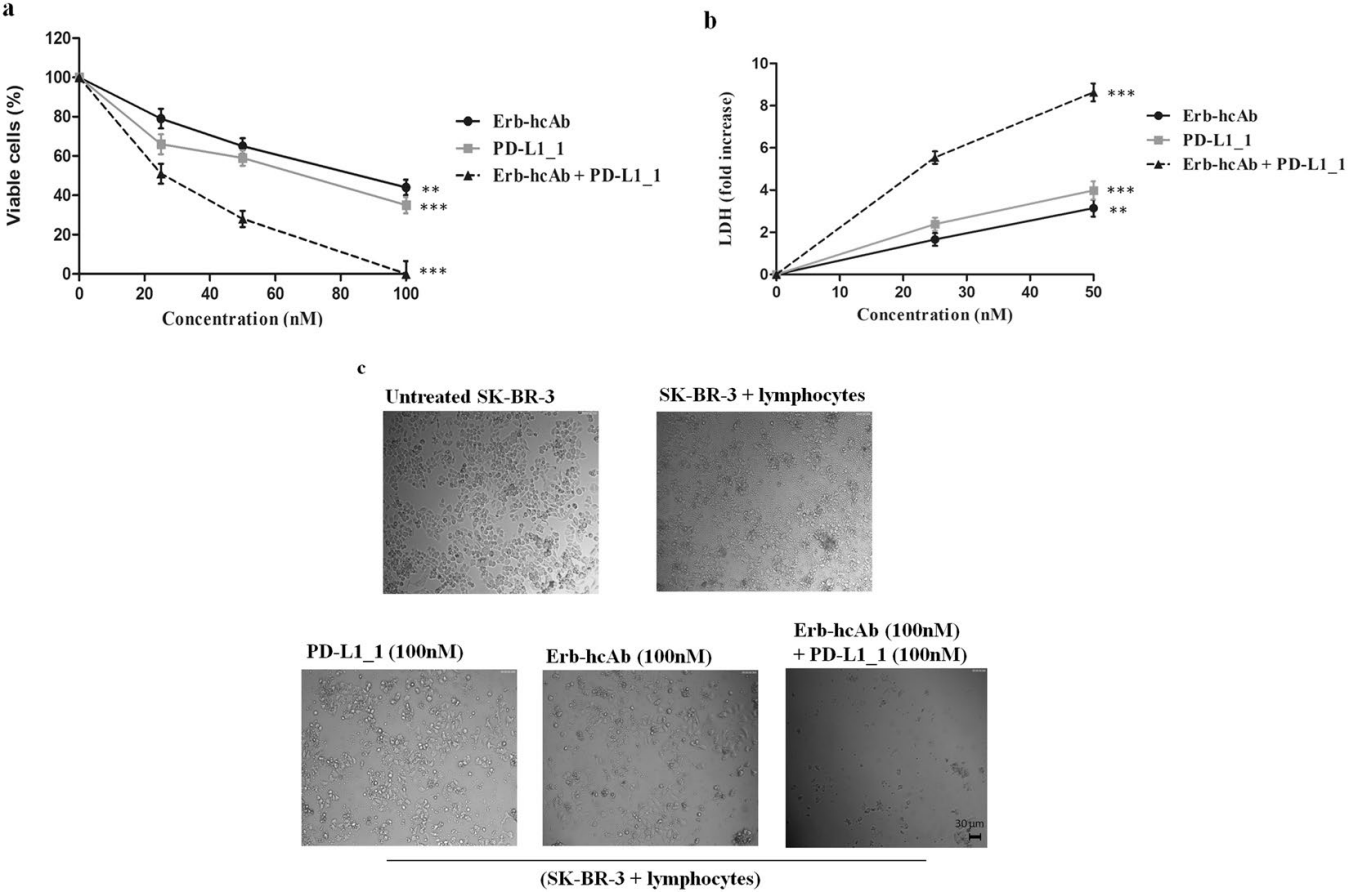
Cytotoxic effects of Erb-hcAb, PD-L1_1 or their combination on tumor cells in the presence of lymphocytes. (**a**) SK-BR-3 cells were co-cultured with lymphocytes (effector: target ratio 10:1), and treated for 24 hours with Erb-hcAb (balck curve, circles) or PD-L1_1 (grey curve, squares) mAbs used alone, or in combination (dashed curve, triangles), at the indicated concentrations. SK-BR-3 cell survival is expressed as percent of viable treated cells with respect to untreated cells. (**b**) SK-BR-3 cell lysis, measured by the LDH release after the incubation with Erb-hcAb or PD-L1_1 mAbs, used alone or in combination at the indicated concentrations. The levels of LDH are expressed as a fold change with respect to the level detected in untreated cells, used as a negative control. (**c**) Images of SK-BR-3 cells treated with each mAb or with the combination of the antibodies tested at the highest concentration (100 nM) in the presence of lymphocytes. The untreated cells in the presence of lymphocytes were used as a negative control. Error bars depicted means ± SD. P values for the indicated mAbs relative to untreated cells, are: ***P ≤ 0.001; **P < 0.01. Scale bar = 30 μm.

The efficacy of the combinatorial approach was then further tested on co-cultures of SK-BR-3 or JMTI-1 tumor cells with hPBMCs to exploit also the effector functions of Erb-hcAb (ADCC mediated by Fc) and the inhibitory effects of PD-L1_1 mAb in the interaction of PD-1/PD-L1^21,38^. To this aim, we treated SK-BR-3 or JMTI-1 tumor cells with each antibody or with their combination at the concentrations of 25–100 nM in the absence or in the presence of hPBMCs (effector: target ratio 10:1) for 24 hours at 37 °C. As shown in Fig. 9, the presence of lymphocytes, as expected, potentiated the antitumor effects of both mAbs on SK-BR-3 cells.

However, the strongest effects were again observed when the two antibodies were used in combination, leading to total inhibition of cancer cell survival when the mAbs were tested at the highest dose of 100 nM (Fig. 9). An unrelated IgG4 isotype antibody was used as a negative control (data not shown).

The cytotoxic effects on target cells were also analyzed in a parallel assay, in which we evaluated the levels of LDH release from treated cells, as a measure of cell lysis^39^. Figure 9b shows that the highest level of LDH release was observed in the supernatant of cells treated with the combination of the two mAbs in the presence of lymphocytes.

Similar potentiated effects were observed when PD-L1–1 was tested in the presence of lymphocytes on JIMT-1 cells, showing additive effects on cell survival and lysis (LDH release) when it was used in combination with Erb-hcAb (data not shown).

## Discussion

Cancer immunotherapy, mediated by both antibodies and immune effector cells, is becoming a precious strategy to overcome the limits of the conventional therapies.

The comprehension of the mechanisms underlying T-cell proliferation and regulation improved the efficacy of the therapeutic approaches with the identification of antibodies agonistic for co-stimulatory or antagonistic for inhibitory receptors to enhance the potential of the immune-based cancer therapy.

Here we investigated the anti-tumor effects on breast cancer cells of a novel anti-PD-L1 antibody, PD-L1_1, generated from a large repertoire of fully human antibodies specific for many T-cell immune checkpoints (ICs) obtained by phage display technology, and capable of inducing T cell activation, cytokine secretion and antitumor effects *in vitro* and *in vivo*^9^.

Indeed, PD-L1 has been recently reported as a potential target for breast cancer, due to its high expression in both ErbB2-positive and Triple Negative Breast Cancer. A high proportion of PD-L1-positive tumors are infiltrated with PD-1-positive lymphocytes and, more interestingly, PD-L1 is expressed not only on T cells but also on breast cancer cells, thus it can be considered as a potential co-target for breast cancer treatments^26,27^.

We found that the novel PD-L1_1 mAb inhibited the growth of breast tumor cells expressing PD-L1 even in the absence of T-cells, thus confirming that PD-L1 plays also an additional role in tumor cells in promoting cell proliferation. We investigated also the effects of two high affinity variants obtained from PD-L1_1 (Cembrola *et al*., Rapid affinity maturation of novel anti-PD-L1 antibodies by a fast drop of the antigen concentration and FACS selection of yeast libraries, submitted for publication, 2019), and we found that they inhibited tumor cell viability more efficiently than the parental PD-L1_1. These results can be explained by considering that PD-L1 has been indeed reported in literature to induce tumor cell proliferation by increasing the levels of Ki-67, p-ERK, p-JNK and p-P38^11,12^, even though there are not yet clear evidences on a complete specific pathway downstream PD-L1.

Thus, we further investigated the anti-tumor potential of PD-L1_1 by evaluating its effects on intracellular pathways downstream PD-L1 in tumor cells. We demonstrated for the first time that the novel anti-PD-L1 mAb (PD-L1_1) can affect the phosphorylation of Erk, JNK and P38 in tumor cells. The high affinity variants of PD-L1_1 showed even more potent effects on the intracellular MAPKs, accordingly with their higher affinity for PD-L1 and their higher *in vitro* antitumor efficacy, thus confirming that these intracellular proteins are indeed regulated by PD-L1 on tumor cells. Further evidences supporting the association of PD-L1 with the mentioned pathways in tumors has been provided by the *in vitro* effects of Atezolizumab on p-Erk and p-JNK. The same activity was confirmed *in vivo* by using PD-L1_1 and a commercially available antibody recognizing the murine PD-L1. Unfortunately, this *in vivo* study was not carried out also with PD-L1_1 high affinity variants as they lost cross-reactivity with mouse PD-L1 after the affinity maturation process (see Fig. 4).

Currently, ongoing clinical trials in cancer patients include immunotherapy based on immunomodulatory antibodies used in combination with chemotherapy or anti-TAA mAbs^8,25^. On the basis of these considerations and of the results relative to the anti-tumor efficacy of the novel isolated PD-L1_1 mAb, we investigated the inhibitory effects of PD-L1_1 on tumor cells in combinatorial treatments with the anti-ErbB2 antibody, Erb-hcAb, capable of inhibiting tumor cell growth *in vitro* and *in vivo*^21^.

Thus, the anti-ErbB2 antibody, Erb-hcAb, was combined with PD-L1_1 mAb for the treatment of ErbB2-positive breast cancer cells, showing that the combinatorial treatment inhibits the growth of tumor cells more efficiently than when they are used as single agents on different tumor cell lines, including the aggressive Trastuzumab-resistant JIMT-1 cells. Even stronger anti-tumor effects were obtained when PD-L1_1 was used in combination with Erb-hcAb on breast cancer cells in the presence of lymphocytes. Indeed, we observed a total inhibition of cancer cell survival when the combination of the mAbs was tested at the dose of 100 nM.

Altogether, these results shed light on the role of PD-L1 in cancer cells and suggest that PD-L1_1 and its high affinity variants can become powerful antitumor weapons that can be used alone or in combination with other drugs, to achieve more potent antitumor effects.

## Methods

### Antibodies and human recombinant proteins

The following antibodies were used: anti-human PD-L1 human mAb [9]; anti-human PD-L1 humanized mAb Atezolizumab (InvivoGen, San Diego, USA); HRP-conjugated anti-human IgG (Promega, Madison, USA); HRP-conjugated anti-human IgG (Fab’)2 goat monoclonal antibody (Abcam, Cambridge, UK); HRP-conjugated anti-rabbit immunoglobulin from goat antiserum (Thermo Fisher Scientific, Meridian Road, USA). Anti-human p-Erk rabbit polyclonal antibody; anti-human p-P38 rabbit polyclonal antibody; anti-human p-JNK rabbit polyclonal antibody; anti-human Cleaved Caspase-3 rabbit polyclonal antibody (all from Cell Signaling, Danvers, USA); Erb-hcAb, human anti-ErbB2 compact Antibody^21^. Anti-mouse PD-L1 (clone 10F.9G2, BioXcell, West Lebanon, USA). The following recombinant proteins were used: human PD-1/Fc (R&D Systems, USA); IFN γ (Peprotech, USA).

### Cell cultures

MDA-MB-231 and JIMT-1 breast cancer cells were cultured in Dulbecco’s Modified Eagle’s Medium (DMEM, Gibco, Life Technologies, Paisley, UK). MCF-7 cells were cultured in Modified Eagle’s Medium (MEM, Gibco, Life Technologies, Grand Island, USA). SK-BR-3 mammary cells and CT26 colon cancer cells were cultured in Roswell Park Memorial Institute 1640 Medium (RPMI 1640, Gibco, Life Technologies, Paisley, UK). Cell lines were purchased from the American Type Culture Collection (ATCC) and cultured in humidified atmosphere containing 5% CO_2_ at 37 °C.

The medium used for JIMT-1 cell line culture was supplemented with 7.5% (vol/vol) heat-inactivated fetal bovine serum (FBS, Sigma); the other media were supplemented with 10% (vol/vol) heat-inactivated fetal bovine serum (FBS, Sigma, USA). All the media were used after addition of 50 UI ml^−1^ penicillin, 50 µg ml^−1^ streptomycin, 2 nM L-glutammine (all from Gibco, Life Technologies, Paisley, UK).

### Enzyme-Linked Immunosorbent Assays (ELISA)

To compare the binding affinities of PD-L1_1 and its variants,, ELISA assays were performed on chimeric PD-L1/Fc protein coated at 5 μg/mL on NuncTM flat-bottom 96-well plates (Thermo Fisher Scientific, 3596). in a solution of 0.05 M NaHCO_3_ for 72 hours at 37 °C. After blocking of the coated 96-well plates with 5% nonfat dry milk in PBS for 1 hour at 37 °C, the purified mAbs were added at increasing concentrations to the plates in 2.5% nonfat dry milk in PBS and incubated for 2 hours at room temperature by gently shaking. After extensive washes with PBS, the plates were incubated with HRP-conjugated anti-human IgG (Fab’)2 goat monoclonal antibody (Abcam, ab98535) for 1 hour, washed again and incubated with TMB reagent for 10 min before quenching with an equal volume of 1 N HCl.

To confirm the binding specificity of PD-L1_1, cell ELISA assays were performed on PD-L1-positive or PD-L1-negative breast cancer cells. Cell ELISA assays were performed by plating the cells in round-bottom 96-well plates (2•10^5^ cells for each well) and incubating them with increasing concentrations (0.5–200 nM) of mAb in 2.5% nonfat dry milk for 2 hours at room temperature with gentle agitation. After the incubation with the primary antibodies, extensive washes were carried out with PBS, then the plates were incubated with an appropriate HRP-conjugated antibody for 1 hour at room temperature, washed again and incubated with 3,3′,5,5′-tetramethylbenzidine (Sigma-Aldrich, St. Louise, USA) reagent for 10 minutes before quenching with an equal volume of 1 N HCl. Absorbance at 450 nm was measured by the Envision plate reader (Perkin Elmer, 2102, San Diego, USA).

For measuring the level of surface PD-L1 expression on tumor cells, SK-BR-3, MDA-MB-231, JIMT-1 and MCF-7 cells (density of 2 x10^5^) were incubated in triplicates the absence or in the presence of the commercial anti-PD-L1 mAb (G & P Biosciences) in PBS/BSA 3% buffer solution for 75 minutes at RT. After extensive washes with PBS 1X solution, the plates were incubated with anti-human IgG (H + L) HRP-conjugate for 1 hour at RT. Then the plates were treated as described above. Binding values were reported as the mean of at least three determinations obtained in three independent experiments.

### Western blotting for the analysis of intracellular pathways

MDA-MB-231 cells were plated at a density of 4•10^5^ cells/well, SK-BR-3 were plated at a density of 6•10^5^ cells/well, MCF-7 cells were plated at the density of 1•10^4^ cells/well in 6-well plates, and incubated for 16 hours at 37 °C. Cells were stimulated with PD-1/Fc (1 µg/ml) or IFNγ (100 ng/ml) or treated with PD-L1_1, its high affinity variants or Atezolizumab, used at the concentration of 200 nM, and incubated for 72 hours at 37 °C. Cells were scraped and centrifuged at 1200 rpm for 5 minutes; the cell pellets were lysed in a buffer containing 10 mM Tris-HCl (pH 7.4), 0.5% Nonidet-P-40, 150 mM NaCl and 1 mM Sodium orthovanadate (Sigma-Aldrich, St. Louise, USA), in the presence of protease inhibitors (Roche, Indianapolis, USA). After incubation on ice for 20 minutes, the extracts were clarified by centrifugation at 12000 rpm for 15 minutes at 4 °C. Protein concentration was determined by the Bradford colorimetric assay (Sigma-Aldrich, USA) and Western Blotting analyses were performed by incubating the membranes with anti-p-Erk, anti-p-P38, anti-p-JNK, anti-pAkt, anti-Akt or anti-Cleaved Caspase-3 antibodies, followed by the HRP-conjugated secondary antibody.

### Isolation of human peripheral blood mononuclear cells (hPBMCs)

Human PBMCs were isolated, as previously reported^9^, by using Greiner Leucosep tube (Sigma-Aldrich, St. Loiuse, USA) following the manufacturer’s instructions, and frozen in a solution containing 90% FBS and 10% dimethyl sulfoxide (DMSO) until use. Cryopreserved cell vials were gently thawed out by using RPMI 1640 medium supplemented with 1% L-glutamine, 1% CTL-Wash (Cellular Technology Limited, Shaker Heights, USA), and 100 U/mL Benzonase (Merck Millipore, Darmstadt, Germany). The collected hPBMCs were then washed by centrifugation, plated and incubated overnight at 37 °C in R10 medium consisting of RPMI 1640 supplemented with 10% inactivated FBS, 1% L-glutamine, 50 U mL^−1^ penicillin, 50 μg mL^−1^ streptomycin and 1% HEPES (GibcoTM, Thermo Fisher Scientific, Paisley, UK). After an overnight resting, the hPBMCs were collected in phosphate-buffered saline (PBS, Verviers, Belgium), counted by using the Muse® Cell Analyzer (Merck Millipore, Darmstadt, Germany) and resuspended at a density of 1•10^6^ cells/mL.

### Cell viability and Cytolysis assays

SK-BR-3 cells were plated at a density of 1.5•10^4^ cells/well in 96-well flat-bottom plates, and incubated for 16 hours at 37 °C. PD-1/Fc (1 µg/ml) and IFNγ (100 ng/ml) were added in the complete culture medium and incubated for further 72 hours. Viable cells were counted by the trypan blue exclusion test and cell survival was expressed as percent of viable cells in the presence of the drugs under test with respect to negative control cultures grown in the absence of the proteins.

For the evaluation of the effects of mAbs, MDA-MB-231 cells were plated at a density of 7•10^3^ cells/well, SK-BR-3 were plated at a density of 1.5•10^4^ cells/well, JIMT-1 were plated at a density of 5•10^3^ cells/well in 96-well flat-bottom plates, and incubated for 16 hours at 37 °C. Erb-hcAb or PD-L1_1 were added at the concentrations of 50–100 nM, alone or in combination, in the complete culture medium and incubated for 72 hours. Viable cells were counted by the trypan blue exclusion test and cell survival was expressed as percent of viable cells in the presence of the drugs under test with respect to negative control cultures grown in the absence of the proteins.

To test the effects of combinatorial treatments on co-cultures of tumor cells and hPBMCs, the cells were plated in 96-well flat-bottom plates at the density of 1.5•10^4^ cells/well for 16 hours, hPBMCs from healthy donors were added at effector:target ratio 10:1 in the absence or presence of increasing concentrations of Erb-hcAb or PD-L1_1 mAbs, used alone or in combination (25–100 nM), and incubated for 24 hours at 37 °C. Controls included target cells incubated in the absence of effector cells or in the presence of the immunoagents alone.

After the treatment, lymphocytes were removed and adherent cells were washed and counted by the trypan blue exclusion test. Cell survival was expressed as percent of viable cells in the presence of the proteins under test with respect to the untreated cells, used as negative control.

Tumor cell lysis was determined by measuring the release of lactate dehydrogenase (LDH) in the supernatant of co-cultures described above by LDH detection kit (Thermofisher Scientific, Rockford, USA), following the manufacturer’s recommendations. Lysis was calculated by measuring the fold increase of LDH in the presence of each mAb, with respect to the amount present in the supernatant of untreated cells, used as a negative control.

Typically, cell survival and cytolysis values were obtained from at least three independent experiments in which triplicate counts were determined. The images of the cells untreated or treated with each compound or with their combinations were acquired by a Leica Microsystems integrated microscope (DFC320, Cambridge, UK).

### *In vivo* studies on mouse models

Six-weeks old female BalBC mice (Envigo, USA) were used for *in vivo* studies. Mice were challenged with a subcutaneous injection of 2•10^5^ CT26 cells (day 0). Three days after, mice were left untreated (control) or treated with PD-L1_1 (200 µg ip) or anti-mouse PD-L1 (200 µg ip, clone 10F.9G2, BioXcell) administered at day 3, 6 and 10. Tumor growth for individual mice was monitored over time using a digital caliper every 3–4 days up to day 21. Tumor volume was calculated by using the formula: 0.5 × length × width^2^.

Tumors from control group and mice treated with PD-L1_1 were harvested at day 21, subjected to three homogenization cycles (3 minutes at 30 Hz) by using RIPA buffer, containing Protease inhibitors and Na_3_VO_4_, and centrifuged. For the analysis of tumor cells in the absence of infiltrating lymphocytes, tumors from control group and mice treated with α-mPD-L1 were also cut into small pieces and digested at 37 °C with Collagenase I. Cell suspension was filtered through a 70 µm cell strainer and incubated with ACK Lysis solution (Gibco, Grand Island, USA). After a last filtration, the cell suspension was placed in a T75 flask at 37 °C, overnight. The day after, adherent cells were washed with PBS, trypsinized, collected and centrifuged. Cell pellets were stored at −80 °C until protein extraction and Western Blotting analyses, performed as described above.

Experiments involving animals have been were approved by the Italian Ministry of Health (Authorizations 213/2016 PR) and have been done in accordance with the applicable Italian laws (D.L.vo 26/14 and following amendments), the Institutional Animal Care and Use Committee of CEINGE and Allevamenti Plaisant SRL.

## Supplementary information

Supplementary Figures

Supplementary Dataset
