# Supplementary material for: Novel Human Anti-PD-L1 mAbs Inhibit Immune-Independent Tumor Cell Growth and PD-L1 Associated Intracellular Signalling
Source: Sci Rep. 2019 Sep 11;9:13125. doi: 10.1038/s41598-019-49485-3 (PMC6739323; doi:10.1038/s41598-019-49485-3)
Supplement: Supplementary file 1 — Supplementary Figures [file 41598_2019_49485_MOESM1_ESM.docx]

**TITLE:** **Novel human anti-pd-l1 mAbs inhibit immune-independent tumor cell GROWTH and pd-l1 associated intracellular signalliNG**

**AUTHORS: Margherita Passariello, Anna Morena D’Alise, Annachiara Esposito, Cinzia Vetrei, Guendalina Froechlich, Elisa Scarselli, Alfredo Nicosia, Claudia De Lorenzo**

**Supplementary Figures**

**
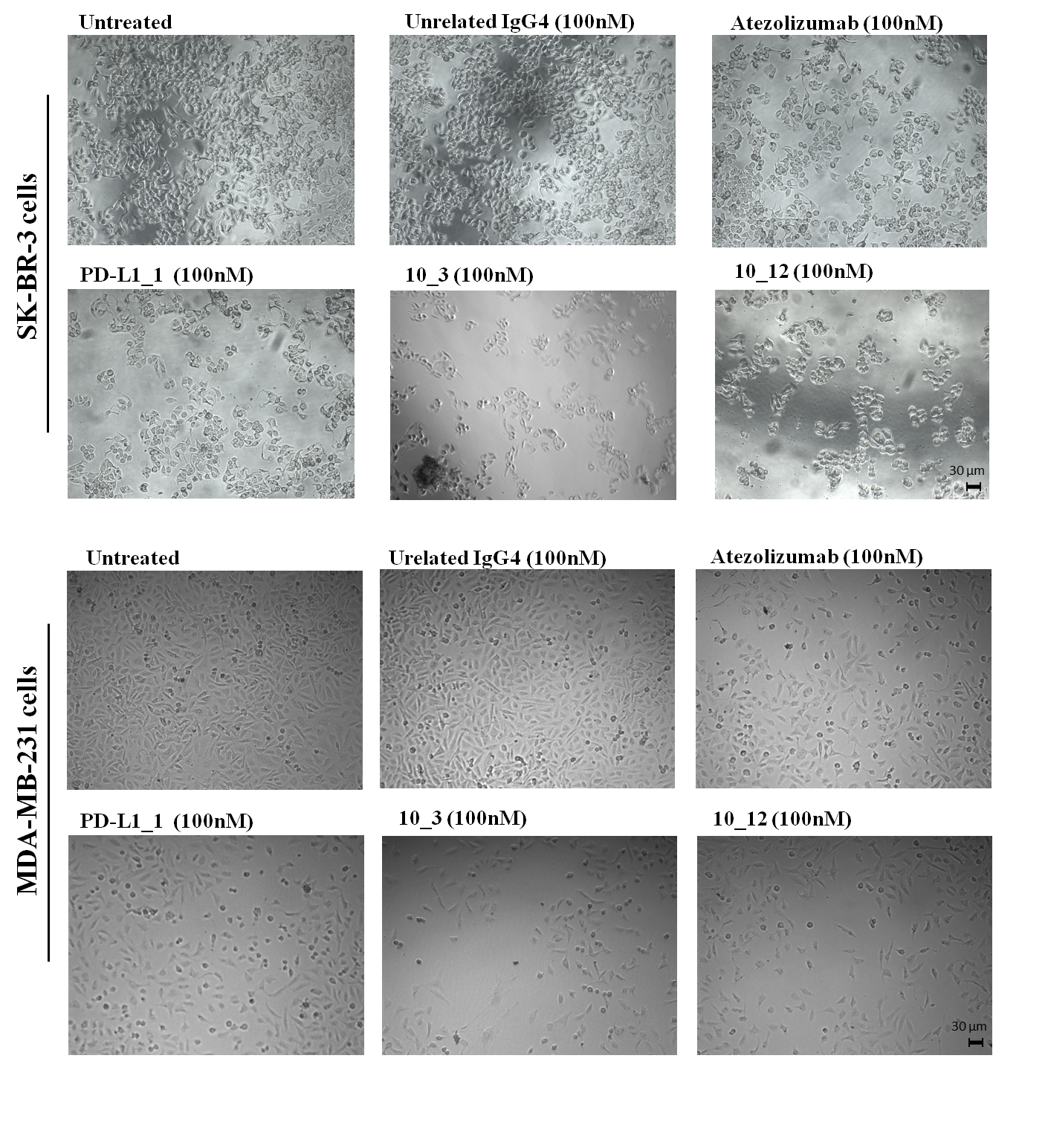
**

**Supplementary Figure S1.** Effects on cancer cells of PD-L1_1 and its high affinity variants. Representative images of breast tumor cells untreated or treated with the indicated antibodies at the concentration of 100 nM. Scale bar = 30 μm.

**
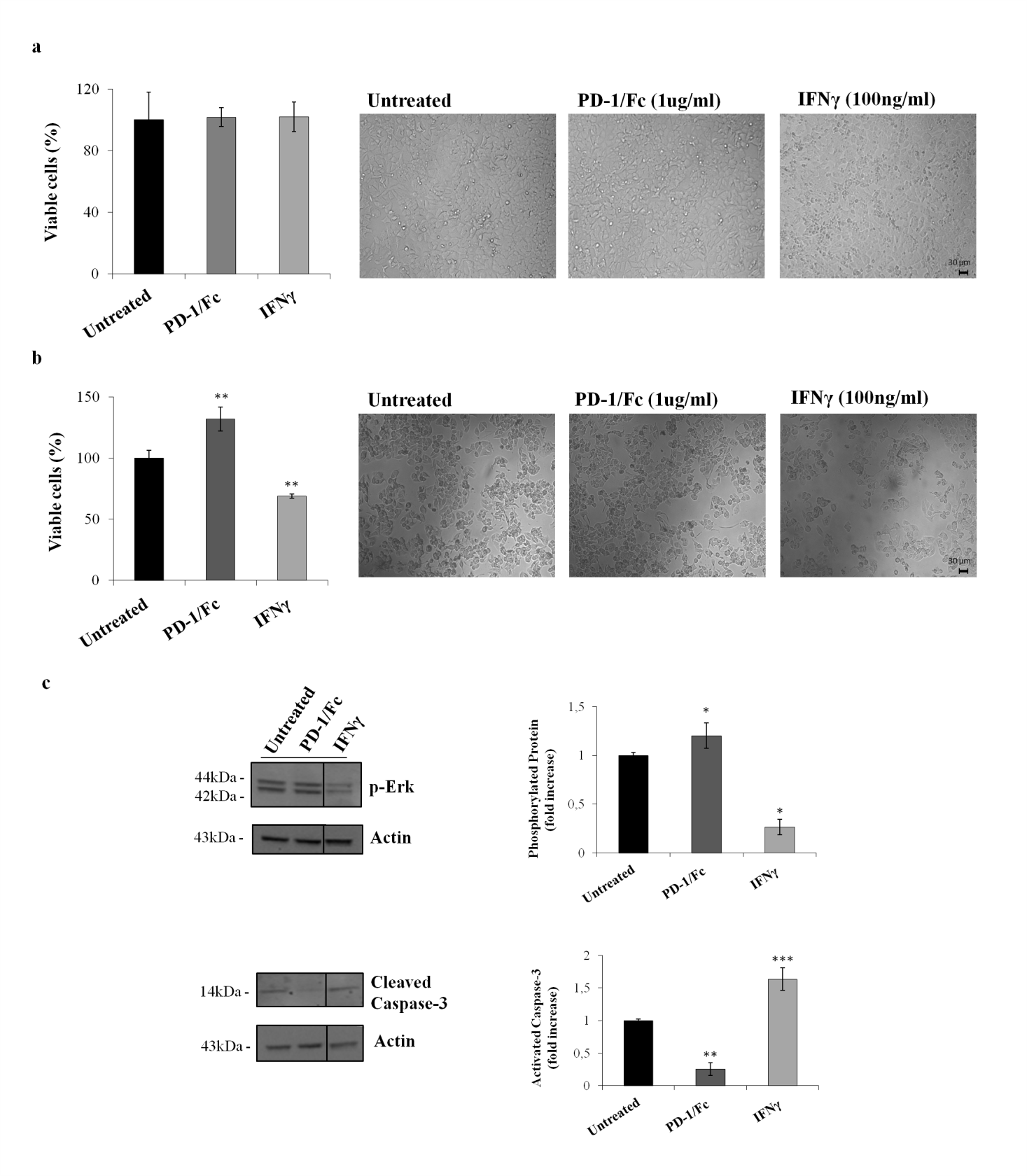
**

**Supplementary Figure S2.** Effects of PD-1/Fc and IFN-γ on tumor cell proliferation and intracellular pathways. Cell survival of PD-L1-negative MCF-7 tumor cells (**a**) or PD-L1-positive SK-BR-3 tumor cells (**b**) treated for 72 hours at 37°C with PD-1/Fc or IFN-γ. The images show some fields of cultures of cells untreated or treated with each indicated compound. Scale bar = 30 μm. (**c**) Western blotting analyses with the indicated antibodies of extracts from SK-BR-3 tumor cells treated with PD-1/Fc or IFN-γ for 72 hours at 37°C. A line has been inserted to indicate distant lanes of the same gel (see Supplementary dataset). Protein levels are also expressed as fold increase with respect to those observed in untreated cells and normalized to actin. Error bars depicted means ± SD. P values for the indicated treatments relative to untreated cells, are: ***P ≤ 0.001; **P < 0.01; *P < 0.05.

**
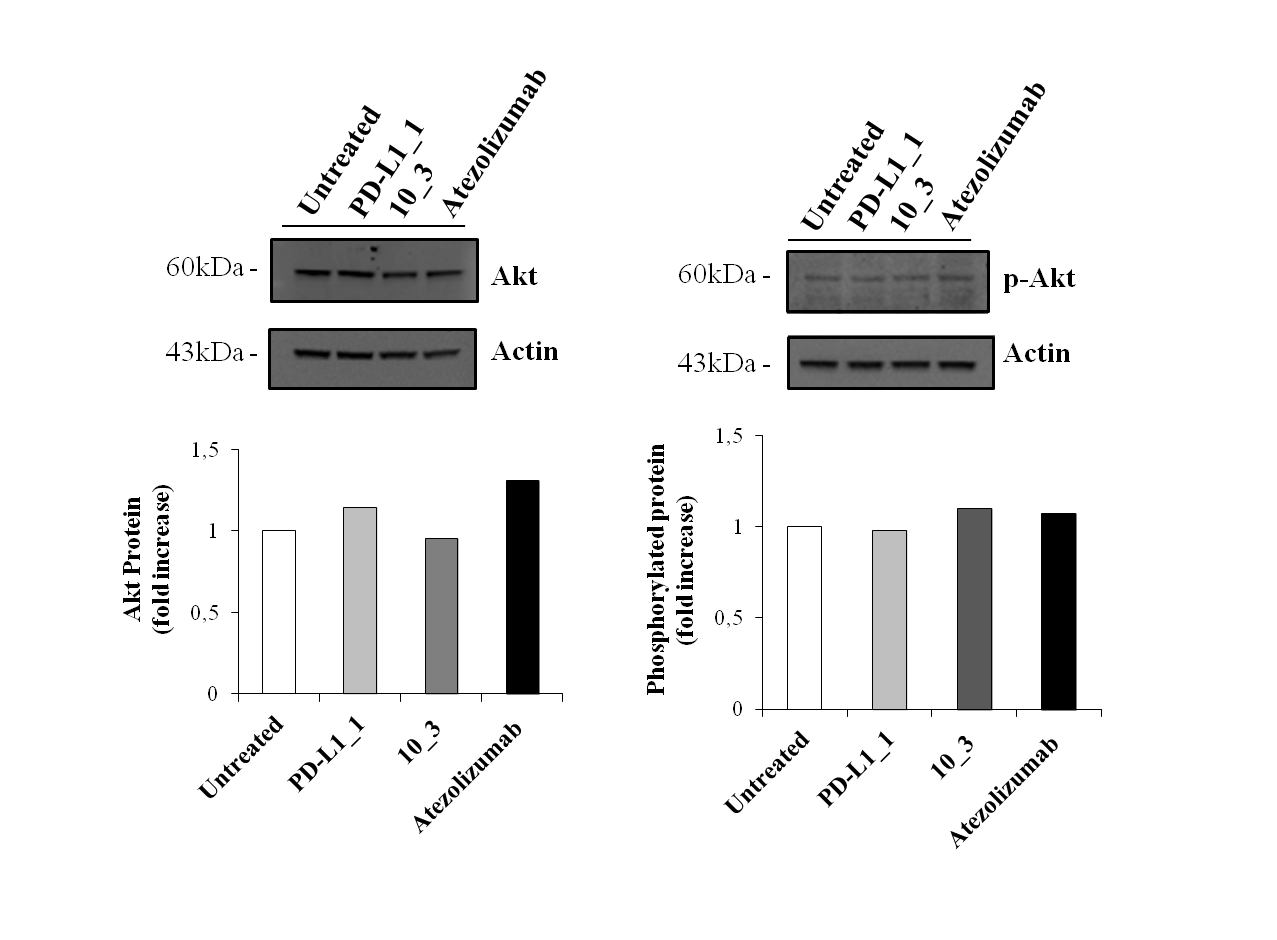
**

**Supplementary Figure S3.** Effects of the anti-PD-L1 mAbs on Akt and pAkt. Western blotting analyses with commercial anti-Akt or anti-pAkt mAbs of cell extracts from SK-BR-3 tumor cells untreated or treated with PD-L1_1, 10_3 or Atezolizumab. Protein levels are also expressed as fold increase with respect to those observed in untreated cells and normalized to actin. Values were reported as the mean of at least three determinations (standard deviations ≤10%).
