# Supplementary material for: Novel Human Anti-PD-L1 mAbs Inhibit Immune-Independent Tumor Cell Growth and PD-L1 Associated Intracellular Signalling
Source: Sci Rep. 2019 Sep 11;9:13125. doi: 10.1038/s41598-019-49485-3 (PMC6739323; doi:10.1038/s41598-019-49485-3)

**TITLE: NOVEL HUMAN ANTI-PD-L1 mAbs INHIBIT IMMUNE-INDEPENDENT TUMOR CELL GROWTH AND PD-L1 ASSOCIATED INTRACELLULAR SIGNALLING**

**AUTHORS: Margherita Passariello, Anna Morena D'Alise, Annachiara Esposito, Cinzia Vetrei, Guendalina Froechlich, Elisa Scarselli, Alfredo Nicosia, Claudia De Lorenzo**

## Supplementary Dataset

## Representative images of Western Blotting analyses reported in the main Manuscript and Supplementary Information files.

**Full-length blot of Figure 5**

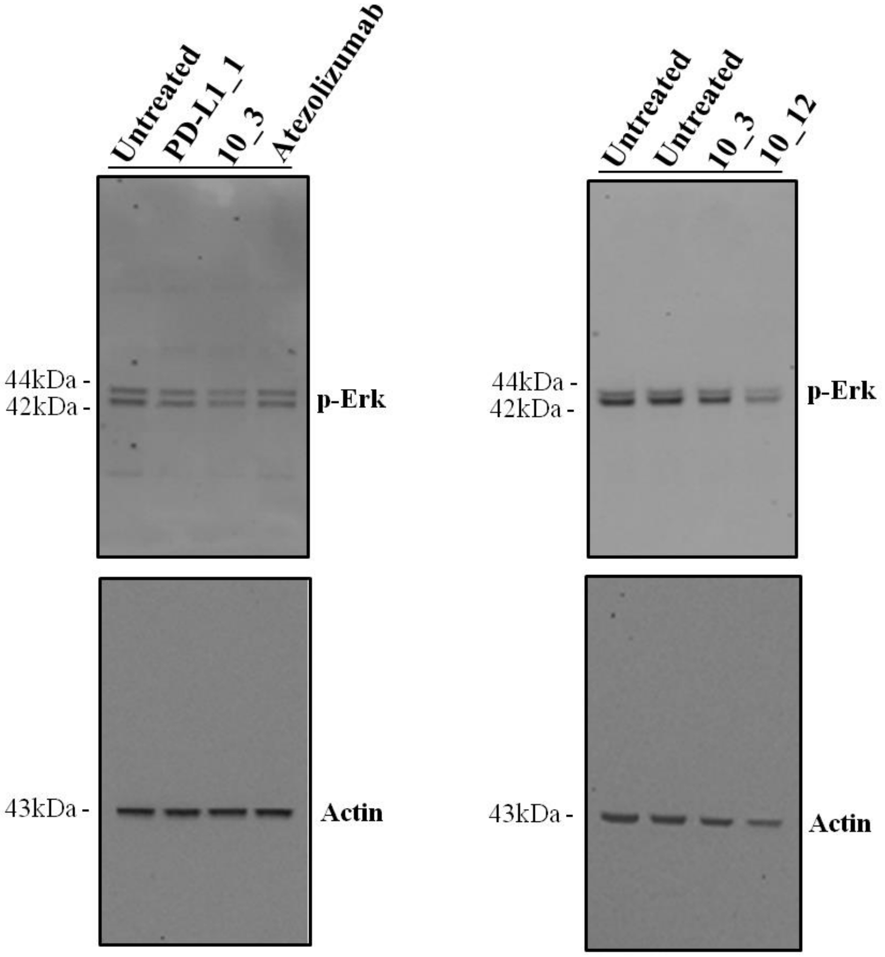

**Full-length blot of Figure 5**

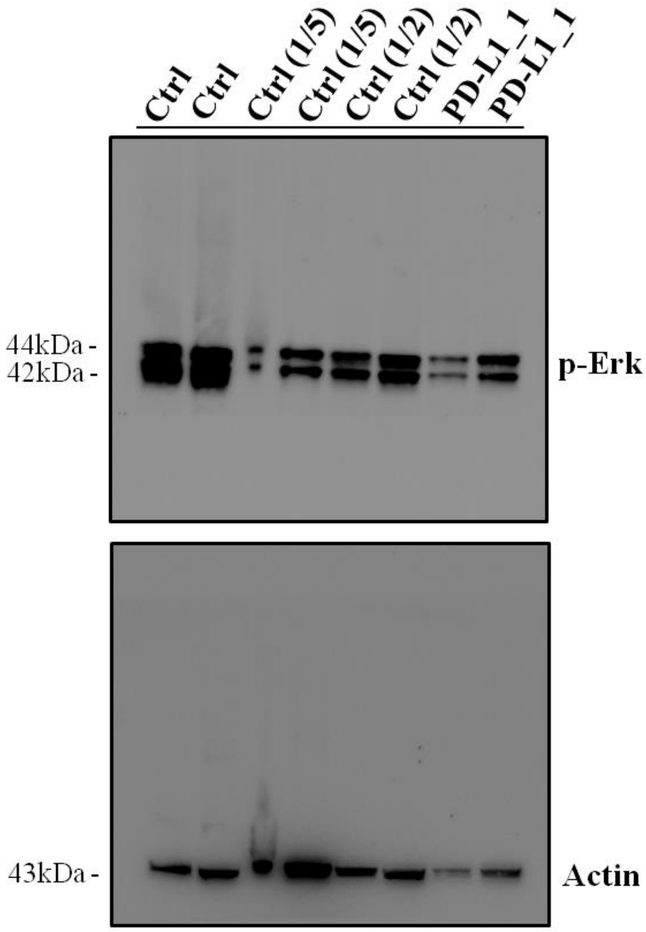


**Full-length blot of Supplementary Figure S2**

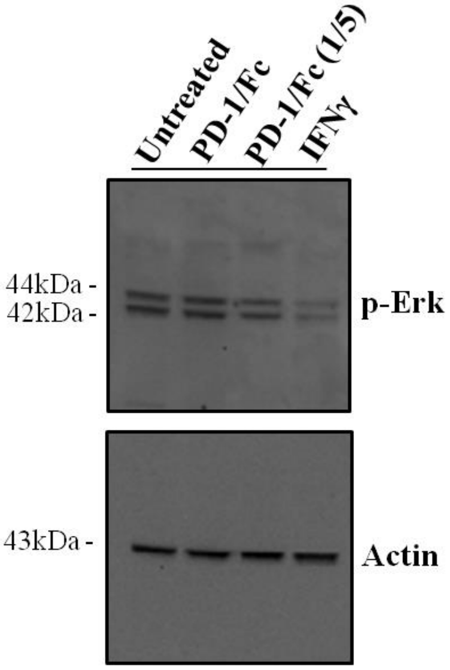


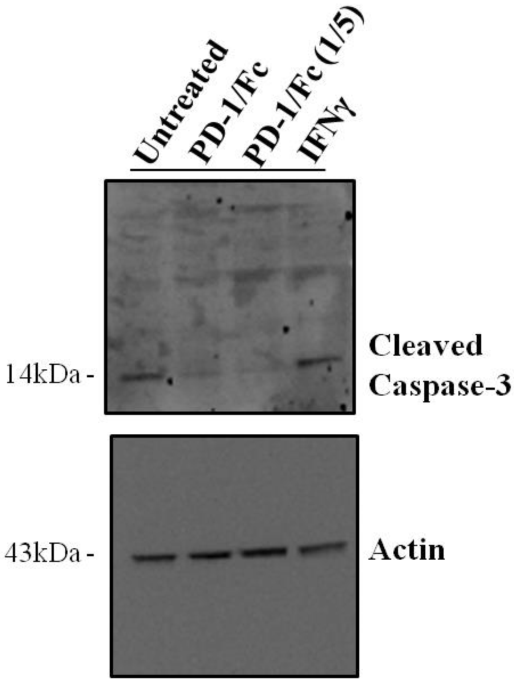

Supplement: Supplementary file 2 — Supplementary Dataset [file 41598_2019_49485_MOESM2_ESM.pdf]
